# Strongest grip on the rod: tarsal morphology and attachment of Japanese pine sawyer beetles

**DOI:** 10.1186/s40851-017-0076-5

**Published:** 2017-09-08

**Authors:** Dagmar Voigt, Takuma Takanashi, Kazuko Tsuchihara, Kenichi Yazaki, Katsushi Kuroda, Remi Tsubaki, Naoe Hosoda

**Affiliations:** 1 Surface & Adhesion Science Group, Research Center for Structural Materials (RCSM), National Institute for Materials Science (NIMS), 1-1, Namiki, Tsukuba, Ibaraki, 305-0044 Japan; 2 Institute for Botany, Technische Universitaet Dresden, D-01062 Dresden, Germany; 3 Forestry and Forest Products Research Institute, Tsukuba, Ibaraki 305-8687 Japan; 4 Department of Information Science, Tohoku Gakuin University, Sendai, Miyagi 981-3193 Japan; 5 Japan Agency for Marine-Earth Science and Technology, Yokosuka, Kanagawa Japan

**Keywords:** Aboreal locomotion, Adhesion, Cerambycidae, Safety factor, Tarsal setae, Traction force

## Abstract

**Background:**

Plant-dwelling beetles overcome challenging attachment hurdles by means of ellaborated, tarsal attachment devices, which are frequently equipped with hairy adhesive pads. While the tarsal functional morphology has been intensively studied in leaf and ladybird beetles during the last decades, longhorn beetles (Cerambycidae) have been widely neglected in experimental studies on insect attachment. However, they are known to strongly hold on to plant substrates, using tarsi that are densely covered with adhesive setae. In the present study, we examined the Japanese pine sawyer beetle, *Monochamus alternatus*, which lives in an arboreal habitat, as an exemplary case. Traction force of male and female beetles on (1) flat and (2) cylindrical, rod-shaped glass was measured. The substrates are further called (1) glass plate and (2) glass rod.

**Results:**

Both male and female *M. alternatus* bear ribbon-shaped tarsal adhesive setae with broadened, ultrathin terminals. They release a distinct amount of adhesion-mediating fluid after each step. The beetles never walked on the vertically oriented glass plate. However, they performed well on the horizontal plate, as well as on the horizontally and vertically oriented glass rod. Males generated higher forces and safety factors (traction force devided by the body weight) than females, reaching up to 206 mN traction force and a safety factor of 32 on the horizontal glass rod. Thus, they are able to carry their remarkable body weight (ca. 300 mg) throughout a rather long life span.

**Conclusions:**

We assume the adaptation of *M. alternatus* to the attachment on convex and rod-shaped structures to be present in its natural habitat. This longhorn beetle species achieves a proper hold through a combination of interacting parameters: appropriate body alignment and two-sided leg grasping, and generation of normal and friction forces by tarsal pads. This smart attachment system gives rise to the reconsideration of the ecological and evolutionary background, and provides insights for the development of bio-inspired technical grippers.

## Background

Insect attachment has attracted scientific attention for centuries. In recent decades, advances have been made in the understanding of the functional tarsal morphology and physical details in beetles (Coleoptera), mainly focused on leaf beetles (Chrysomelidae) and ladybird beetles (Coccinellidae), culminating in the development of beetle-inspired adhesive tape [1]. Longhorn beetles (Cerambycidae), however, have been widely neglected, despite a number of impressive features, including their remarkable antennae, their particular, elongated body shape, long and slender legs, and tarsi with broadened “felty soles” (e.g. [2]). Similar to other beetles, they bear adhesive setae, as previously confirmed for representatives of the subfamilies Lepturinae (*Rhagium mordax* De Geer, *Grammoptera ruficornis* Fab.) and Cerambycinae (*Clytus arietis* L.) [3], which correspond to hairy insect attachment systems as defined by Beutel and Gorb [4].

In the present study, we focused on the Japanese pine sawyer beetle, *Monochamus alternatus* Hope, as a model species, a member of the longhorn beetle subfamily Lamiinae, representatives of which are associated with Angiospermae [5]. Twenty-seven host plant species are reported, all belonging to Pinaceae, Coniferales [6–8]. The xylobiont genus *Monochamus* is distributed throughout the world, feeding on dead or dying wood/trees (larvae) and on bark of one-to-two-year old pine twigs (maturing adults), and transmitting phytoparasitic nematodes [5, 7, 9, 10]. The adult pine sawyer beetles live for up to 100–125 days; females live longer than males. Their flight and walking activity varies depending on weather conditions, age/physiological conditions, and daytime. Adults disperse by walking and short-range flights in the pine canopy (7–40 m per week) [6]. These beetles rely on tarsal attachment and locomotion while (1) exploring new feeding and oviposition sites, (2) feeding, (3) oviposition, (4) intraspecific competition, and (5) mating. In order to explore new feeding and oviposition sites, Japanese pine sawyer beetles attach and walk on various substrates, such as bark and leaves, each with different geometry, material and surface properties. While feeding on pine bark, the beetles grasp a twig between legs and move the head down and upwards, chewing on plant tissue. Oviposition involves a complex procedure that combines zigzag walks, gnawing of bark to form a wound, turning the body to lay the eggs and cover them with a jelly, and rubbing the oviposition sears with the abdominal tip [8, 11]. During copulation, the male mounts the back of the female, turning and orienting to the same direction as the female, and grasps the female metathorax with forelegs. Thus, the 1st, 2nd and 3rd tarsomeres of forelegs are attached to the female metasternum on both body sides. At this posture, the female is “half-mounted”, and walks non-stop with the male clasping her metathorax. During the copulatory bond of 23–390 min, the male forelegs and midlegs grasp the female while the hindlegs hold onto the host plant surface (e.g., [8]). Moreover, males compete for females, which may involve fighting with antennae, guarding a mate, multiple mating, or male-to-male mounts [8, 12, 13]. All such activities require a proper hold, which is also needed to lay eggs.

Live adults carry a considerable body weight of about 300 mg, and females are heavier and larger than males [7]. The body length and width of females average 21 and 7 mm, respectively, while those of males average 20 and 6 mm [14, 15]. Thus, the relatively large and heavy Japanese pine sawyer beetles cope with various shapes and surfaces of substrates under different life conditions in an aboreal habitat, overcoming mechanical challenges on cylindrical twigs or branches. Therefore, they are equipped with conspicuously long, slim legs, with an aspect ratio of 23, corresponding to three-fourths the body length and about 25 times the body width (D.V., personal observation). Long, grasping extremities are characteristic for aboreal locomotion, providing a firm grip and stable hold in discontinuous habitats in any position [16].

The question of how tarsi contribute to the attachment ability in *M. alternatus* is thus of interest. Here we combined microscopic and experimental studies in order to characterize the tarsal morphology and attachment of longhorn beetles. The natural surfaces of two common Japanese pine species, as well as elytra, tarsi, and footprints of *M. alternatus*, were visualized using cryo-scanning electron microscopy (cryo-SEM). Additionally, traction force measurements with male and female longhorn beetles on flat and cylindrical, rod-shaped glass were carried out in order to obtain widely comparable data and to check the significance of tarsal setae in overall attachment while excluding claw interlocking with substrates. These findings lead to new insights in evolutionary, ecological and/or biomimetic aspects of insect attachment.

## Methods

### Insects and plants

Beetles were obtained from dead pine trees, *Pinus thunbergii* Parl. and *Pinus densiflora* Siebold et Zucc. (Pinaceae) infested with larvae of *M. alternatus* at the Forestry and Forest Products Research Institute (36° 07′ 00″ N, 140° 03′ 00″ E) and its Chiyoda Experimental Station (36° 11′ 7″ N, 140° 12′ 58″ E) in Ibaraki Prefecture, Japan. Beetles were kept in ventilated plastic boxes (250 ml volume), at 22.3 ± 2.5 °C temperature and 29.1 ± 6.2% relative humidity and 8 h photoperiod during the study. A few twigs (about 100 mm long) of *P. thunbergii* and *P. densiflora* were provided as diet and replaced ad libitum every several days. Four-to-five-years-old seedlings of *P. thunbergii* and *P. densiflora* equipped with twigs (1600 mm long, diameter of lower trunk 25 mm, diameter of upper trunk 20 mm, diameter of twigs 10 mm), were freshly cut at the winter state in the field of the Forestry and Forest Products Research Institute. For analyses and experiments, adults 1–2 months after emergence from pupae were used.

### Ocular and microscopic observations

Postures of the body, feet and legs of longhorn beetles were observed and documented by using a digital camera Canon EOS Kiss X5 (Canon Inc., Japan), a stereomicroscope Zeiss Discovery.V12 (Carl Zeiss Microscopy GmbH, Jena, Germany), and an inverted research light microscope Nikon Eclipse Ti-S (Nikon Corp., Tokyo, Japan).

To visualize beetle tarsi and natural substrates of *M. alternatus* at a high magnification, metal holders with attached samples were frozen in liquid nitrogen, transferred to the preparation chamber, maintained at −185 °C, sputter-coated with an about 10-to-20 nm layer of gold, and examined in a frozen state in the cryo-SEM JEOL JSM-6510 equipped with a cryo unit and an evaporation system and the Software SEM Controller Interface 2.23 (JEOL Ltd., Tokyo, Japan) at 3 kV and −120 °C (see [17] for further details). Samples were mounted on holders using Tissue-Tek® O.C.T.™ Compound mounting fluid (Sakura Fine Technical Co., Ltd., Tokyo, Japan). Dimensions of beetle tarsi were estimated using the software SigmaScan Pro 5 (SPSS Inc., Chicago, USA). Non-sputter-coated traces of adhesion-mediating fluid, left by attached and subsequently detached feet on metal-covered resin surfaces were observed according to Gorb [18] and Gorb et al. [19]; however, resin droplets were prepared from Agar low viscosity resin R1078a-d kit (Agar Scientific Ltd., Stansted, Essex, UK) and coated with a ca. 15 nm layer of gold prior to microscopic examination.

### Traction force measurements

Traction force measurements were conducted with adult longhorn beetles (1–2 months after emergence from pupa) on a flat and a rod-shaped glass substrate. Cleaned micro glass slides (thickness 0.9–1.2 mm, 76 × 26 mm, S-7213, Matsunami glass Ind., Ltd., Japan), a custom-made glass plate, and custom-made, 100-mm long glass rod of 20 mm diameter were used. In order to clean them, the glasses were successively rinsed with acetone, ethanol and Aqua Millipore water, and immediately dried with compressed air. The FSE of test substrates laid in the range of those of natural substrates of *M. alternatus* (see the Additional file 1 for the measurement method and Additional file 1: Figures S4, S5 for data); on the flat glass slide: 52.0 mN*m^−1^ (polar component: 33.4 mN*m^−1^, disperse component: 18.6 mN*m^−1^) and on glass rod: 50.5 mN*m^−1^ (polar component: 34.6 mN*m^−1^, disperse component: 15.9 mN*m^−1^). Accordingly, no impact of FSE was expected comparing the glass substrates in experiments.

Prior to tests, beetles were weighed using the balance AND GR-202 (A&D Company Ltd., Japan). Then, they were attached to a thin high-grade polyester sewing thread (Schappe spun no. 90, FUJIX Ltd., Kita-ku, Kyoto, Japan) by droplets of 1:1 bees wax-colophonium mixture at the beetle dorsum. The free end of the thread was connected to a force transducer FORT25 (25 g, serial 83271 046) combined with the BIOPAC System MP100 and software ACQ Knowledge 3.7.3 (World Precision Instruments, Inc., Sarasota, FL, USA). Five males and five females were tested on both glass plate and rod. Three runs per each individual were recorded at a sample rate of 200 per second and averaged for further evaluations. Laboratory climate conditions were kept at 22.3 ± 2.5 °C temperature and 29.1 ± 6.2% relative humidity. Statistical evaluation was carried out using the software SigmaStat 3.1.1 (Systat Software, Inc., Richmond, CA, US).

## Results

### Morphological diversity of natural substrates

The natural substrates of *M. alternatus* appear to be highly variable, including plant cuticle (leaves), bark and wood, as well as insect cuticle (elytra). The host plant surface properties strongly vary depending on the site and plant organ (Additional file 1: Figure S2, Table S3). Beetle elytra are irregularly covered with numerous microtrichia (Additional file 1: Figure S3): (1) longer ones on the thorax and the elytron fissure, and (2) shorter ones on the distal and lateral elytron. Along the lateral margin, a sharp edge is situated (Additional file 1: Figure S3B, E, H). Between the microtrichia, hair-free patches are found. The elytra, microtrichia, and hair-free patches are partially covered with epicuticular grease (Additional file 1: Figure S3 I, J, arrow heads). The FSE of natural substrates of *M. alternatus* ranged from 25 to 65 mN*m^−1^, involving more disperse and less polar components (Additional file 1: Figures S4, S5).

### Body posture, tarsal morphology and secretion

**Fig. 1:**
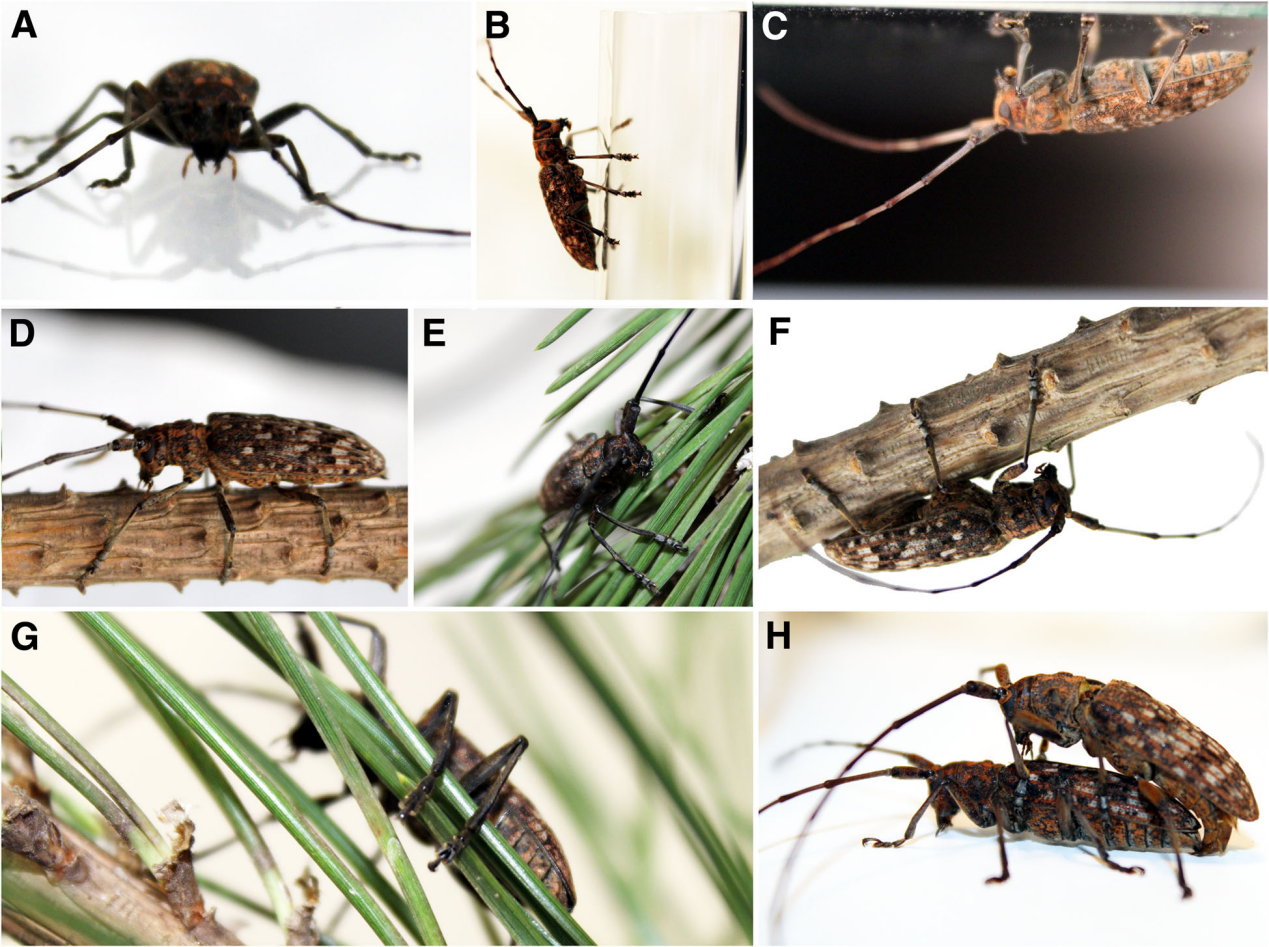
*Monochamus alternatus* attached to different substrates at different positions: horizontal (**a**, **d**, **e**), vertical, upwards (**b**), and ceiling (**c**, **f**). **a**, **c**. Glass plate. **b**. Glass rod. **d**, **f**. Pine twigs (bark). **e**. Pine needles (leaves). **g**. A female clamps a bulk of several needles shaping some larger rod-shaped structure between their legs. **h**. A male grasping a female during copulation. Tarsi of fore- and middle legs adhere to the elytra, while the hind legs keep in contact with the substrate, but not with female’s body

In our study, males and females were observed to attach to any part of host plants at any position during walking, resting, feeding, oviposition, grooming, and copulation (Fig. 1), including bark of twigs and stems, plant leaves and longhorn beetle’s body (during copulation). Obviously, even wood particles, caused by the feeding of beetles on twigs, do not interfere the attachment of beetles.

**Fig. 2:**
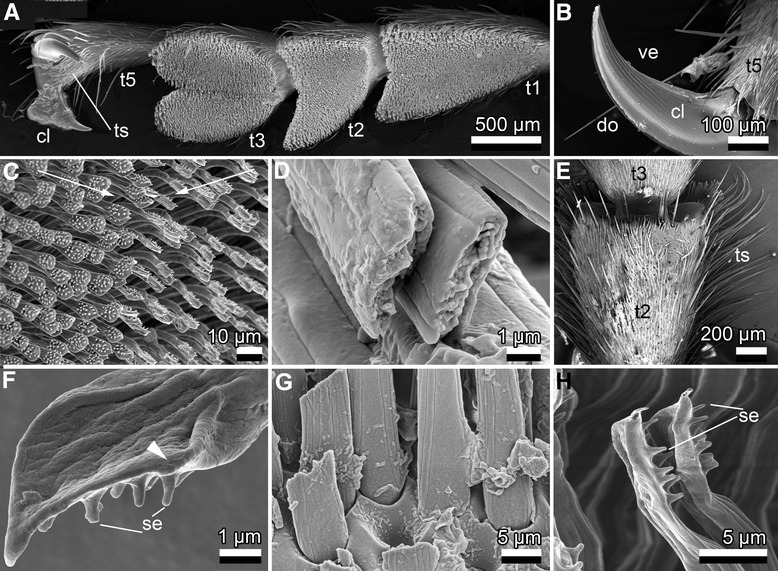
Cryo-SEM micrographs of tarsal structures of female *Monochamus alternatus.* **a**. Ventral view of a female’s foretarsus. Note the distinctly bilobed pad in the 3rd tarsomere. **b**. A single part of the paired claws, equipped with sharp edges ventrally and dorsally (female). **c**. Terminally spatulate, adhesive setae on the first tarsomere of a female’s foreleg. The arrows indicate the opposite, anisotropical orientation of setal terminals, with adhesive surfaces facing each other, according to the bilobed pad of the first tarsomere as mentioned for (**a**). **d**. Cross section of setae, showing their flattened, ribbon-like shape (female). **e**. Dorsal view of male’s second tarsomere, laterally bearing numerous long tactile setae. **f**. Ventro-lateral view of the setal terminal, illustrating the tapered tip and spindle-shaped adhesive area surrounded by a ridge (arrow head), and dorsally situated setules (female). **g**. Broadened, flattened basal shafts of setae (female). **h**. Lateral details of terminals of spatula-shaped female’s tarsal adhesive setae, showing 5–10 setules (se) at their dorsal side, suggesting the avoidance of coagulation of the extremely thin (ca. 0.3 μm) terminals. t1, 1st tarsomere; t2, 2nd tarsomere; t3, 3rd tarsomere; t5, 5th tarsomere; ts, tactile setae; cl, claw; do, dorsal; se, setule; ve, ventral. Note that t4, the 4th tarsomere is hidden and not shown in the image

Tarsi of *M. alternatus* are composed of five flattened, broadened segments (tarsomeres 1–5) (Fig. 2a; Additional file 1: Figure S1, Table S1); however, the fourth one is hidden. Tarsomeres are about 1230 μm (1st), 765 μm (2nd), 783 μm (3rd), 1264 μm (5th) long, and about 760 μm (1st), 846 μm (2nd), 866 μm (3rd), 423 μm (5th) wide (*n* = 1 female). The third tarsomere appears distinctly bilobed. The fifth tarsomere bears paired, curved, 604 μm long claws with 8 μm wide tips (*n* = 4, one male and one female pooled together; Fig. 2b). Tarsomeres 1–3 are all covered with adhesive hairs (setae) (Fig. 2a, c, d, f-h, Additional file 1: Table S2).

No sexual dimorphism in the shape and size of setal terminals was observed. The setae possess broadened, spatulate, tapered terminals (Fig. 2c, f, h) and flattened, ribbon-shaped shafts (Fig. 2d, g). The ventral terminals are 8 μm long, 5 μm wide, 0.3 μm thick, and appear spindle-shaped and soft (*n* = 20, 2 males and 2 females pooled together; Fig. 2f). Dorsally they are covered with 5–10, 1.2 μm long, and 0.3 μm thick setulae (*n* = 20, two males and two females pooled together; Fig. 2f, h).

The setal shaft is patterned by longitudinal striae. A slight gradient in the length of setae, increasing from the proximal (37 μm) to the distal (80 μm) part of each tarsomere, was observed (*n* = 1, male). The average length of a centrally situated seta is 73 μm, the width is about 4 μm at the base, 5 μm at the terminal (see Additional file 1: Table S2, for all data). The terminal spatula is 8 μm long, 5 μm wide, and 0.3 μm thick, corresponding to an area of 26 μm^2^ (*n* = 1 male). For example, on a single female foreleg, the total number of adhesive setae was estimated to be about 23,600 (1st tarsomere: 5300; second tarsomere: 4900; third tarsomere: 13,400; *n* = 1). Thus, the potential contact area may be estimated at 613,600 μm^2^ for a single tarsus and 3,681,600 μm^2^ (3.7 mm^2^) for all tarsi of six legs pooled together.

The setae are obviously “oppositionally”/anisotropically oriented, corresponding to the lobes of the tarsomeres (Fig. 2c, arrows). Their dorsal side, bearing setulae, always points laterally, while the apparently soft, adhesive area faces the middle of the tarsomere.

**Fig. 3:**
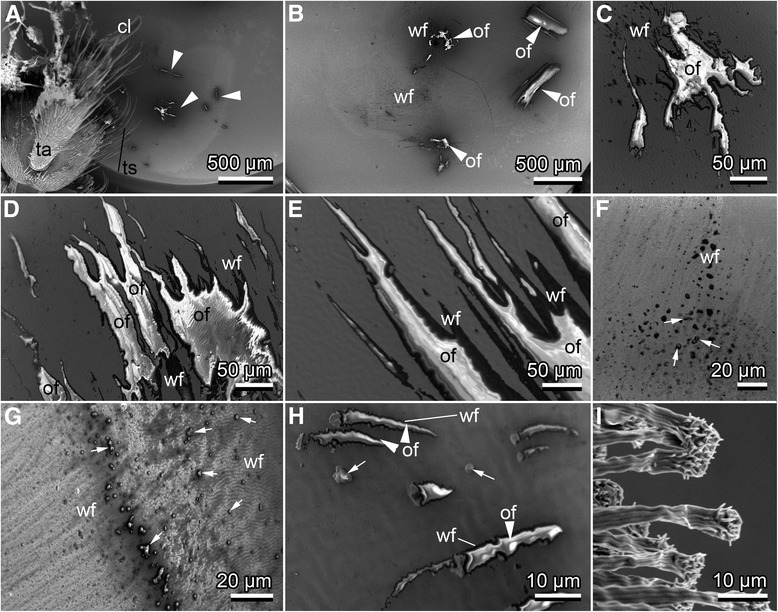
Footprints left by tarsal setae of *Monochamus alternatus* after bringing them in contact with and subsequently remove them from a gold-covered resin surface visualized by cryo-SEM; male (**a**, **b**, **d**, **e**, **h**, **i**); female (**c**, **d**, **f**, **g**.). **a**. 3rd to 5th tarsomeres close to their print on the surface from where they were detached during freezing and vacuum procedure. **b–h**. Residues of footprints. **i**. Terminal adhesive setae on a male foreleg’s tarsus for comparison with the dimension of footprints (**a**-**h**). cl, claw; ta, tarsus; ts, tactile setae; wf, presumably watery fluid; of, presumably oily fluid; arrows indicate contamination with wood particles; arrow heads point to oily fluid residues

Interestingly, contaminating particles were found between setal shafts, but not on the adhesive terminals. Residues of adhesive fluid (small droplets) were observed at a few sites on tarsal setae. *M. alternatus* left distinct traces of adhesion-mediating fluid, frequently mixed with particles (Fig. 3). The fluid appeared liquid and wetting. Dark/black areas of evaporated fluid (watery, Fig. 3, wf) and shiny, charged areas of fluid residues were detected (Fig. 3, of). Evidently, the fluid spread widely, forming ultrathin layers on the hydrophilic test surface. Single droplets, released by single tarsal adhesive setae, mostly coagulated to larger drops.

### Traction forces

Males were significantly lighter (411.8 ± 68.03 mg) than females (520.4 ± 114.43 mg) (mean ± sd, *n* = 10 per sex, t-test, *t* = 2.6, *P* = 0.02).

On the horizontal glass plate, traction forces ranged between 35 and 117 mN in males, as well as 34 and 102 mN in females (Table 1). On the horizontal glass rod, males and females generated higher traction forces of 78–196 mN and 42–149 mN, respectively. Considering all 6 legs and adhesive setae in contact (total area of 3.7 mm^2^, assumed for both sexes) with the surface, and supposing the maximum measured traction to be dominated by friction force, a lateral tenacity (shear stress) in setal terminals of *M. monochamus* may be roughly estimated as 35 kPa in males and 20 kPa in females on the horizontal glass rod.

**Table 1:** Traction forces [mN], mean ± sd (*n* = 5), generated by adult *Monochamus alternatus* on different substrates

Substrate	Male	Female
Horizontal glass plate	59.3 ± 32.59 a	66.0 ± 26.94 a
Vertical glass plate	<4.0*	< 5.1*
Horizontal glass rod	127.7 ± 43.71 b	74.1 ± 42.93 ab
Vertical glass rod	63.0 ± 20.73 ab	29.9 ± 10.80 b

Letters indicate statistical differences in male or female traction forces between test substrates according to Kruskal-Wallis one-way ANOVA on ranks, Tukey-test, *p* < 0.05, males: *H*_2,13_ = 6.7, *p* = 0.034; females: *H*_2,13_ = 7.3, *p* = 0.025; *, no attachment

**Fig. 4:**
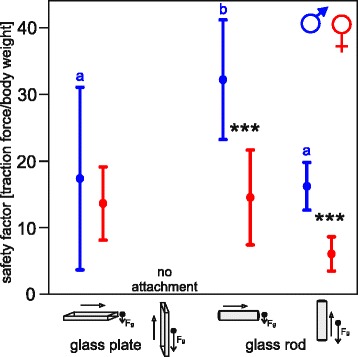
Safety factors generated by male and female *Monochamus alternatus* on a glass plate and glass rod; scatterplot showing column means and standard deviations. Attachment failed on vertically oriented, flat glass. Arrows with unfilled heads point to the direction of traction, and arrows with filled heads point to the direction of acting gravity. Dots show means with standard deviations (*lines*). The asterisks mark statistical differences between males (*blue*) and females (*red*) on horizontal flat glass: Mann-Whitney test, *T* = 26, *p* = 0.841, on horizontal glass rod: *t*-Test, *t* = −3.5, *p* = 0.009, and on vertical glass rod: *t*-Test, *t* = −4.6, *P* = 0.002; different letters indicate statistical differences between substrates in males (one-way ANOVA, F_2,13_ = 4.2, *P* = 0.04, Fisher LSD method). Values not differ significantly between substrates in females (one-way ANOVA, F_2,13_ = 3.7, *P* = 0.056)

For comparison, safety factors (forces related to the body weight) were incorporated (Fig. 4). Males performed significantly better than females, and significantly best on the horizontal glass rod (2.2 times higher value than on the glass plate). In females, no statistical differences in safety factors between substrates were found. However, the mean safety factor, generated by females on the glass rod, was 1.1 times higher than on the glass plate. The ratio of safety factors generated on horizontal and vertical glass rod was 1.9:1 in males and 2.1:1 in females.

## Discussion

### Attachment ability

Given the broad variety of natural substrates, including leaf cuticle, bark, wood, wood particles, and elytron cuticle, one may assume a universal attachment ability of *M. alternatus*, resistant to a broad range of surface textures, chemistry, geometry, and contamination. The well wetting, large residues of footprints support this assumption, and further indicate some self-cleaning effect of the feet by the secretion, as particles were frequently detected inside the fluid traces. Similar hypotheses have been previously proposed for the function of tarsal secretion in smooth adhesive systems of stick insects [20]. Cryo-SEM images of the spreading and coagulating fluid secretion of *M. alternatus* let assume watery and oily components, indicated by dark traces of evaporated fluid (watery, Fig. 3, *wf*), and shiny, charged areas (oily, Fig. 3, *of*), respectively. Additionally, we suggest the adhesive setae of *M. alternatus* to be highly flexible and adaptable to a variety of substrates, even bark and wood. This is in contrast to leaf beetles, which predominantly adhere to and walk on surfaces of leaves or leaf stems. Considering the rather long life span of *M. alternatus* adults, they rely on a sufficient, long-term tarsal function.

**Fig. 5:**
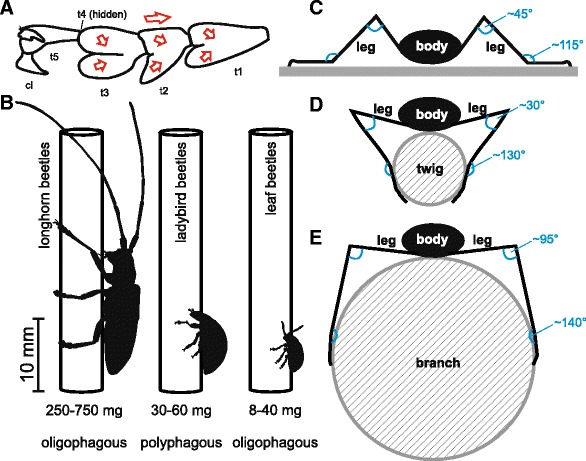
Schematic drawings illustrating several aspects in longhorn beetle attachment. **a**. Directions of shearing (*arrows*) on the ventral tarsus, according to the alignment of adhesive setae (see Fig. 2 for comparison). t1–t5, tarsomeres; cl, claw. **b**. Comparison of body dimension and posture between longhorn, ladybird, and leaf beetles grasping a rod-shaped substrate. **c–e**. The longhorn beetle body posture on a flat and a rod-shaped substrate having different diameter. *Blue lines* and letters indicate the angles between leg segments

Traction force values of male Japanese pine sawyer beetles on the glass plate were up to 4-to-7 fold higher than that of male dock leaf beetles *Gastrophysa viridula* De Geer and Colorado potato beetles *Leptinotarsa decemlineata* Say (Coleoptera, Chrysomelidae) ([21, 22], D.V., unpublished data), which may be attributable to the efficient adhesive structures (Figs. 2, 5a) and a higher muscle mass in longhorn beetles [23]. *M. alternatus* is rather large and heavy compared to other species of beetles, which were included in scientific studies on attachment so far (Fig. 5b). Accordingly, tarsal dimensions were 2–3 times larger in *M. alternatus* compared to that of, e.g., the about 20 times lighter, smaller dock leaf beetles *G. viridula* [24]. Attachment ability is also reflected by the number of tarsal setae, e.g., on a female fore tarsus: in total, 23,600 in *M. alternatus* and 1100 in *G. viridula* [24]. In contrast to numerous leaf beetles, ladybird beetles, and longhorn beetles belonging to the subfamily Lepturinae [3], no sexual dimorphism in the shape of adhesive setae in *M. alternatus* was observed. This is in accordance with representatives of Cerambycinae [3], and maybe explained by the seta-covered elytra that are not sufficient to create a contact with male-specific (discoid) setae during copulation. Differently, in beetle species, bearing male-specific tarsal setae, females are equipped with smooth, non-structured elytra (e.g., *L. decemlineata*, [21]).

Flattened, ribbon-shaped adhesive setae with spindle-shaped, spatulate terminals consistently cover the ventral 1st to 3rd tarsomeres in both male and female Japanese pine sawyer beetles. According to Stork [3], these ultra-thin structures are prevented from coagulation by tiny setules, which distinctly cover the dorsal terminal side. Adhesive setae occur anisotropically arranged, facing opposite each other on the bilobed tarsomeres (Fig. 5a). This arrangement indicates the directionality and let expect a simultaneous double-peeling of many spatulae at the level of contralateral pads of a single tarsomere within one foot, resulting in a hierarchical multiple peeling configuration at the level of the whole beetle [25, 26]. The tape-like shape of both the flattened setal shafts and terminals slightly differs from those found in previously studied beetles [3]. Flattened adhesive tapes facilitate material strength and efficient peeling dynamics and, thus, a proper detachment resistance of setae. The setal tenacity of 35 kPa in males and 20 kPa in females resembles previously reported values for tarsal setae in syrphid flies (15–35 kPa; [27]). Since a wider film generates a higher peeling force [28], the numerous, relatively broad setae in *M. alternatus* result in a long peeling line and 3–7 times stronger pulling forces compared to those of leaf beetles, reaching up to 102 mN on flat glass and 206 mN on the horizontal glass rod. Considering the corresponding safety factors of 17 and 32, respectively, leaf beetles perform better than longhorn beetles on flat glass, generating safety factors of 146 (male *G. viridula*) and 43 (male *L. decemlineata*) (D.V., personal observation). Much higher forces and safety factors for *M. alternatus* are expected in natural habitats, while additionally applying and interlocking claws with surface irregularities. The 604-μm long claws, having a tip diameter of 8 μm, are 2–6 times longer and 2–4 times wider than in previously studied leaf beetles (D.V., personal observation). Thus, paired claws in longhorn beetles probably provide a strong support, anchor and lever, particularly on rough and fissured surfaces. Such effects of surface roughness on attachment have to be proved in future experiments. The shape of a single claw not let deduce the sufficient grasping of structures, although the claw is equipped with sharp inner and outer edges. However, similar to an anchor, claws may strongly interlock with counterparts when acting pairwise in concert.

### Arboreal attachment

Japanese pine sawyer beetles rely on a proper tarsal attachment exploring their arboreal habitat while feeding, oviposition, intraspecific competition, mating, migrating, and escaping from enemies.

In the traction force experiment, forces acting on the insect were applied in a lateral direction to the surface. *M. alternatus* generated forces, walking and pulling horizontally on the flat glass and glass rod. Thus, traction forces correspond to the friction between beetle pretarsi and the substrate; however, muscle force cannot be neglected. Longhorn beetles did not attach and pull on a vertical flat glass surface. In contrast, on a vertically oriented glass rod, specimens walked upward and pulled, generating considerable forces and safety factors, up to 17 in males and 7 in females. Under these conditions, weight force (gravity) is acting at the center of mass. The situation is comparable to climbing on twigs or branches or mounting the back of a vertically walking female. Here, a higher portion of adhesion than friction forces should provide a suitable hold, so far claw interlocking is prevented as it was the case in our study. Comparing safety factors, generated on the horizontal and vertical glass rod, friction:adhesion ratios are 1.9:1 and 2.1:1 for male and female *M. alternatus*, respectively. Thus, friction is the larger component in the attachment of longhorn beetles while holding in a vertical position on rod-shaped substrates. This shows no evident differences from that in previously studied insect species equipped with smooth adhesive systems on flat surfaces in centrifugal force experiments; e.g., sawfly larvae *Rhadinoceraea micans* Klug on Plexiglas (3.1:1; [29]) adult codling moths *Cydia pomonella* L. on Plexiglas (1.6:1; [30]), and specialized ants on Perspex (2.2:1; [31]). Interestingly, hairy pads of blowflies *Calliphora vomitoria* L., horizontally and vertically pulling on glass, reached a friction:adhesion ratio of maximum 8.3:1, however, generated safety factors 2–5 times lower than that in *M. alternatus* [32].

We suppose an even higher attachment ability of *M. alternatus* under different conditions. As shown by Gladun and Gorb [33] for different, climbing insect species, belonging to the orders Orthoptera, Heteroptera, Coleoptera, and Hymenoptera, the whole tarsus is involved in gripping a cylindrical substrate, depending on the diameter of the latter. Similarly, our observations revealed that *M. alternatus* grasps a 20-mm thick cylindrical substrate, using the 1st to 3rd tarsomeres, holding its tibio-femoral joints perpendicular and the tibio-tarsal joints at a wide angle (Fig. 5e). Exemplarily, *M. alternatus* was tested on a glass rod, corresponding to the dimension of naturally occupied substrates, such as pine branches. Considering the hierarchical functionality of feet, legs, and body, one may suggest a complex combination of the gripping mechanism and locomotion dynamics is required for successful attachment of longhorn beetles in the arboreal habitat. One recent study revealed that frogs use friction and normal forces of roughly a similar magnitude for holding on to cylindrical objects. When climbing a non-adhesive surface, the compressive forces between opposite legs nearly doubled, indicating a stronger clamping grip [34]. Such a clamping is also conceivable in *M. alternatus* under similar conditions, supported by the overall body posture (Fig. 5c-e) and the ratio of body length and width to leg length and width (Fig. 5a), whose range in *M. alternatus* appears to be typical for arboreal locomotion [16]. The relatively large feet close around perches, allowing the generation of a torque preventing the beetles from toppling sideways. Commonly, gripping strength and grasp forces are greater in longer feet, resulting in higher walking speed of aboreal living animals [35].

At the tarsal level, the ribbon-shaped, adhesive setae support the gripping of rod-shaped substrates. Interestingly, similar shaped adhesion-promoting setae are found in the grasping feet and the ventral subdigital and distal caudal skin of chameleons and dwarf gecko digits [35–37]. The flattened, blade-like plates have been described to be capable of being adpressed to the surface through compression. The analogy in the shape of adhesive setae in both chameleons and longhorn beetles, which both occupy aboreal habitats, lets us conjecture that ribbon-shaped, flattened setae are related to the arboreal life style and grasping of rod-shaped substrates. In this regard, recent studies with chameleons confirmed that habitat use drives the evolution of gripping organ morphology through its effects on performance and clade-specific adaptations [35, 36]. This aspect merits investigation in longhorn beetles (Cerambycidae), which together with Megalopodidae, Orsodacnidae, Oxypeltidae, and Vesperidae form one of the two lineages evolved within the superfamily Chrysomeloidea [38].

## Conclusions

The present study sheds light on the role of hairy pads in the attachment system of the Japanese pine sawyer beetles, disregarding interlocking mechanisms of claws. Although they possess distinctly larger body weight and dimensions, the generated forces and safety factors are comparable with previously tested insects.

Since high traction forces and safety factors were generated on the cylindrical, rod-shaped substrate in both males and females, we suppose certain adaptation of legs and the tarsal attachment system of *M. alternatus* to convex structures like rods (twigs, stems), and elytra. Correspondingly, beetles were observed walking mainly on twigs, trunks, stems, and pine needles, shaping bundles of needles between their legs. However, they appeared to be less agile on flat substrates.

Future experimental setups should shed light on the attachment to differently rough surfaces, considering both flat and rod-shaped structures, and the impact of various diameters of the latter. We suggest a proper hold of *M. alternatus* to diverse surfaces, because the flattened, ribbon-shaped adhesive setae in combination with the adhesion-mediating fluid seem to be universal in their capability to attach to a variety of substrates. Further knowledge about the chemical composition of the fluid will add to the overall understanding of longhorn beetle attachment.

Our results stimulate a critical re-consideration of maximum attachment forces in insects, which are dependent on the optimum substrate geometry and condition. Moreover, they provide insights into the universal attachment properties of longhorn beetle feet, which may contribute to the development of bio-inspired adhesives for climbing robots [39]. The present findings are of particular interest given that beetle-inspired adhesives to date have focused on examples of static flat-to-flat contacts (e.g. [1]).

## Additional file

Additional file 1: Supplement. (PDF 1218 kb)

## Abbreviations

cryo-SEM: cryo-scanning electron microscopy
FSE: Free surface energy

## References

[1] Gorb S, Varenberg M, Peressadko A, Tuma A (2007). Biomimetic mushroom-shaped fibrillar adhesive microstructure. J R Soc Interface.

[2] Klausnitzer B, Klausnitzer U, Wachmann E, Hromádko Z (2015). Die Bockkäfer Mitteleuropas.

[3] Stork NE (1980). A scanning electron microscope study of tarsal adhesive setae in the Coleoptera. Zool J Linnean Soc.

[4] Beutel R, Gorb SN (2001). Ultrastructure of attachment specializations of hexapods (Arthropoda): evolutionary patterns inferred from a revised ordinal phylogeny. J Zool Syst Evol Res.

[5] Linsley EG (1959). Ecology of Cerambycidae. Annu Rev Entomol.

[6] Kobayashi F, Yamane A, Ikeda T (1984). The Japanese pine sawyer beetle as the vector of pine wilt disease. Ann Rev of Entomol.

[7] Kishi Y (1995). The pine wood nematode and the Japanese pine sawyer. Forest pests in Japan – no. 1.

[8] Zhao BG, Futai K, Sutherland JR, Takeuchi Y (2008). Pine wilt disease.

[9] Kojima T (1931). Further investigation on the immature stages of some Japanese Cerambycid beetles, with notes on their habits. J Coll Agric Imp Univ Tokyo.

[10] Breuning S (1944). Études sur les Lamiares (Col. Ceramb.) Douzième Tribu: Agniini Thomson. Nov Entomol.

[11] Anbutsu H, Togashi K (2000). Deterred oviposition response of *Monochamus alternatus* (Coleoptera: Cerambycidae) to oviposition scars occupied by eggs. Agric For Entomol.

[12] Fauziah A, Hidaka T, Tabata K (1987). The reproductive behavior *of Monochamus alternatus* Hope (Coleoptera: Cerambycidae). Appl Entomol Zool.

[13] Kim G-H, Takabayashi J, Takahashi S, Tabata K (1992). Function of pheromones in mating behavior of the Japanese pine sawyer beetle *Monochamus alternatus* Hope. Appl Entomol Zool.

[14] Ochi K, Katagiri K (1974). Ecological studies on the Japanese pine sawyer, *Monochamus alternatus* HOPE (Coleoptera: Cerambycidae) (II) size and shape of adults in the field populations. J Jpn For Soc.

[15] Ya-Qi G, Yu-Cui X, Xi T, Zhu-Dong L (2015). Body size difference of male and female adults as well as the relationship between the sizes of pupae and adults and the body weight of overwintering larvae in the Japanese pine sawyer, *Monochamus alteratus* (Coleoptera: Cerambycidae). Acta Entomol Sinica.

[16] Schmidt M (2010). Locomotion and postural behaviour. Adv Sci Res.

[17] Yazaki K, Kuroda K, Nakano T, Kitao M, Tobita H, Ogasa MY, Ishida A (2015). Recovery of physiological traits in saplings of invasive *Bischofia* tree compared with three species native to the Bonin Islands under successive drought and irrigation cycles. PLoS One.

[18] Gorb S (2006). Fly microdroplets viewed big: a cryo-SEM approach. Micros Today.

[19] Gorb SN, Schuppert J, Walther P, Schwarz H (2012). Contact behaviour of setal tips in the hairy attachment system of the fly *Calliphora vicina* (Diptera, Calliphoridae): a cryo-SEM approach. Zoology.

[20] Clemente CJ, Federle W (2012). Mechanisms of self-cleaning in fluid-based smooth adhesive pads of insects. Bionisp Biomimetics.

[21] Voigt D, Schuppert JM, Dattinger S, Gorb SN (2008). Sexual dimorphism in the attachment ability of the Colorado potato beetle *Leptinotarsa decemlineata* (Coleoptera: Chrysomelidae) to rough substrates. J Insect Physiol.

[22] Voigt D, Schweikart A, Fery A, Gorb S (2012). Leaf beetle attachment on wrinkles: isotropic friction on anisotropic surfaces. J Exp Biol.

[23] Pedley TJ (1977). Scale effects in animal locomotin.

[24] Bullock JMR, Federle W (2009). Division of labour and sex differences between fibrillar, tarsal adhesive pads in beetles: effective elastic modulus and attachment performance. J Exp Biol.

[25] Pugno NM (2011). The theory of multiple peeling. Int J Fracture.

[26] Heepe L, Raguseo S, Gorb SN (2017). An experimental study of double-peeling mechanism inspired by biological adhesive systems. Appl Phys A Mater Sci Process.

[27] Gorb SN, Gorb E, Kastner V (2001). Scale effects on the attachment pads and friction forces in syrphid flies (Diptera, Syrphidae). J Exp Biol.

[28] Varenberg M, Pugno NM, Gorb SN (2010). Spatulate structures in biological fibrillar adhesion. Soft Matter.

[29] Voigt D, Gorb SN (2012). Attachment ability of sawfly larvae to smooth surfaces. Arthropod Struct Dev.

[30] Al Bitar L, Voigt D, Zebitz CPW, Gorb SN (2009). Tarsal morphology and attachment ability of the codling moth *Cydia pomonella* L. (Lepidoptera, Tortricidae) to smooth surfaces. J Insect Physiol.

[31] Federle W, Baumgartner W, Hölldobler B (2004). Biomechanics of ant adhesive pads: frictional forces are rate- and temperature dependent. J Exp Biol.

[32] Walker G, Yue AB, Ratcliffe J. The adhesive organ of the blowfly, *Calliphora vomitoria*: a functional approach (Diptera: Calliphoridae). J Zool Lond. 1985;205:297-307.

[33] Gladun D, Gorb SN (2007). Insect walking techniques on thin stems. Arthropod Plant Interact.

[34] Endlein T, Ji A, Yuan S, Hill I, Wang H, Barnes WJ, Dai Z, Sitti M (2017). The use of clamping grips and friction pads by tree frogs for climbing curved surfaces. Proc R Soc B.

[35] Herrel A, Tolley KA, Measey GJ, da Silva JM, Potgieter DF, Boller E, Boistel R, Vanhooydonck B (2013). Slow but tenacious: an analysis of running and gripping performance in chameleons. J Exp Biol.

[36] Khannon ER, Endlein T, Russell AP, Autumn K (2013). Experimental evidence for friction-enhancing integumentary modifications of chameleons and associated functional and evolutionary implications. Proc R Soc B.

[37] Russell AP, Baskerville J, Gamble T, Higham TE (2015). The evolution of digit form in *Gonatodes* (Gekkota: Sphaerodactylidae) and its bearing on the transition from frictional adhesive contact in gekkotans. J Morphol.

[38] McKenna DD, Wild AL, Kanda K, Bellamy CL, Beutel RG, Caterino MS, Farnum CW, Hawks DC, Ivie MA, Jameson ML, Leschen RAB, Marvaldi AE, McHugh JV, Newton AF, Robertson JA, Thayer MK, Whiting MF, Lawrence JF, Ślipinski A, Maddison DR, Farrell BD (2015). The beetle tree of life reveals that Coleoptera survived end-Permian mass extinction to diversify during the cretaceous terrestrial revolution. Syst Entomol.

[39] Voigt D, Karguth A, Gorb S (2012). Shoe soles for the gripping robot: searching for polymer-based materials maximising friction. Rob Auton Syst.

