# Supplementary material for: Strongest grip on the rod: tarsal morphology and attachment of Japanese pine sawyer beetles
Source: Zoological Lett. 2017 Sep 8;3:16. doi: 10.1186/s40851-017-0076-5 (PMC5591571; doi:10.1186/s40851-017-0076-5)
Supplement: Additional file 1: — Supplement. (PDF 1218 kb) [file 40851_2017_76_MOESM1_ESM.pdf]

Strongest grip on the rod: tarsal morphology and attachment of Japanese pine sawyer beetles

Supplement

Tarsal morphometry

Table S1 Dimensions of tarsal segments in *Monochamus alternatus* [µm] (for indication of letters see Fig. S1), *n* = 1 for t1-5 (tarsomere 1-5, length and width); mean ± sd, *n* = 4 for cd (claw tip diameter); *n* = 2 for cl (claw mean length)

Site	cd	cl	t5	t5a	t5b	t3	t3a	t3b	t2	t2a	t2b	t1	t1a	t1b
♀ Midleg	8.3 ± 0.85	603.7 ± 88.26	1288.2	261.4	126.7	823.5	697.3	322.5	680.1	718.9	386.7	1151.4	616.7	264.4
♀ Foreleg			1223.9	521.6	191.0	763.7	1001.0	373.3	703.0	918.0	448.4	1263.6	812.0	296.5
♂ Foreleg			1278.6	486.9	177.3	760.4	900.4	331.8	910.7	900.2	373.0	1273.7	851.0	497.1

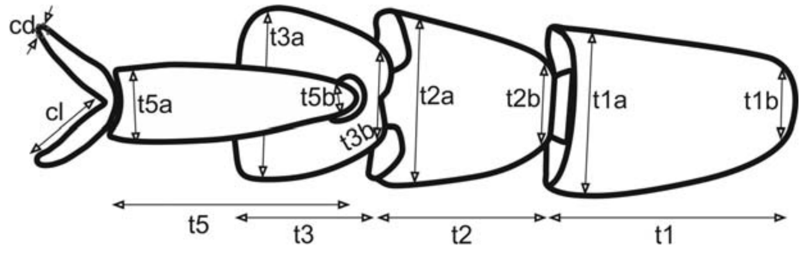

Fig. S1 Scheme of the tarsus of *Monochamus alternatus* and definition of measured dimensions (data provided in Table S1). cd, claw tip diameter; cl, inner claw length; t1-5, tarsomeres 1-5; a, distal width; b, basal width.

Table S2 Estimated dimensions [μm] of several parameters of tarsi in *Monochamus alternatus* (*n* = 20)

**Parameter**	**Mean**	**Sd**	**Measurement site**
Length of proximal, spatula-shaped adhesive setae	37.3	2.93	♀ foreleg and midleg, 3rd tarsomere
Length of central and distal, spatula-shaped adhesive setae	73.1	12.60	♀ foreleg and midleg, 3rd tarsomere
Width of the base of spatula-shaped adhesive setae	3.6	0.54	♀ foreleg and midleg, 3rd tarsomere
Width of the terminal of spatula-shaped adhesive setae	5.4	0.66	♀ foreleg and midleg, 1st, 2nd, and 3rd tarsomere
Thickness of the terminal of spatula-shaped adhesive setae	0.3	0.07	♀ foreleg and midleg, 1st, 2nd, and 3rd tarsomere
Length of the spindle-shaped adhesive area on ventral terminals of spatula-shaped adhesive setae	7.8	1.02	♀ foreleg and midleg, 1st, 2nd, and 3rd tarsomere
Length of setules on terminals of spatula-shaped adhesive setae	1.2	0.29	♀ foreleg and midleg, 1st, 2nd, and 3rd tarsomere
Width of setules on terminals of spatula-shaped adhesive setae	0.3	0.05	♀ foreleg and midleg, 1st, 2nd, and 3rd tarsomere
Length of tactile setae on the 5th tarsomere	110.9	19.83	♀ foreleg and midleg
Width of tactile setae on the 5th tarsomere	4.4	1.13	Width at the middle of setae; ♀ foreleg and midleg
Length of laterally tactile setae in male tarsi	120.6	29.17	♂ foreleg, 2nd, and 3rd tarsomeres
Width of laterally tactile setae in male tarsi	9.1	2.44	Width at the middle of setae; ♂ foreleg, 2nd, and 3rd tarsomeres

Characteristics of host substrates

Table S3 Characteristics of host plant surfaces, to which longhorn beetles *Monochamus alternatus* attach to

Species	Plant organ/site	Description
Pinus densiflora (Fig. S2A, C, E, G, I, K)	Needle of current-year shoot	o epicuticular wax crystals visible (platelets),<br>o rows of stomata, high abundancy of wax crystals in and around stomatal grooves,<br>o longitudinal folds (by cell shape pattern)
	Needle of one-year old shoot	o epicuticular wax crystals altered/abraded – almost not visible
Pinus densiflora (Fig. S2A, C, E, G, I, K)	Shaft of needle (current year)	o distinct longitudinal (flat) ripening/foldings
	Bark of branch	o rough surface, but not fissured
	Bark of upper trunk	o fissured surface, however less fissured than that of lower trunk
	Bark of lower trunk	o fissured, fractal structures, "very rough", cracks and slight "plateaus" alternate
Pinus thunbergii (Fig. S2B, D, F, H, J, K)	Needle of current-year shoot	o a very few sites covered with epicuticular wax crystals visible (platelets),<br>o rows of stomata, high abundancy of wax crystals inside stomatal grooves,<br>o longitudinal folds (by cell shape pattern)
	Needle of one-year old shoot	o epicuticular wax crystals altered/abraded – almost not visible,
	Shaft of needle (current year)	o distinct longitudinal (flat) ripening/foldings
	Bark of branch	o rough surface, but not fissured
	Bark of upper trunk	o rough surface, but not fissured
	Bark of lower trunk	o fissured, fractal structures, "very rough", cracks and slight "plateaus" alternate

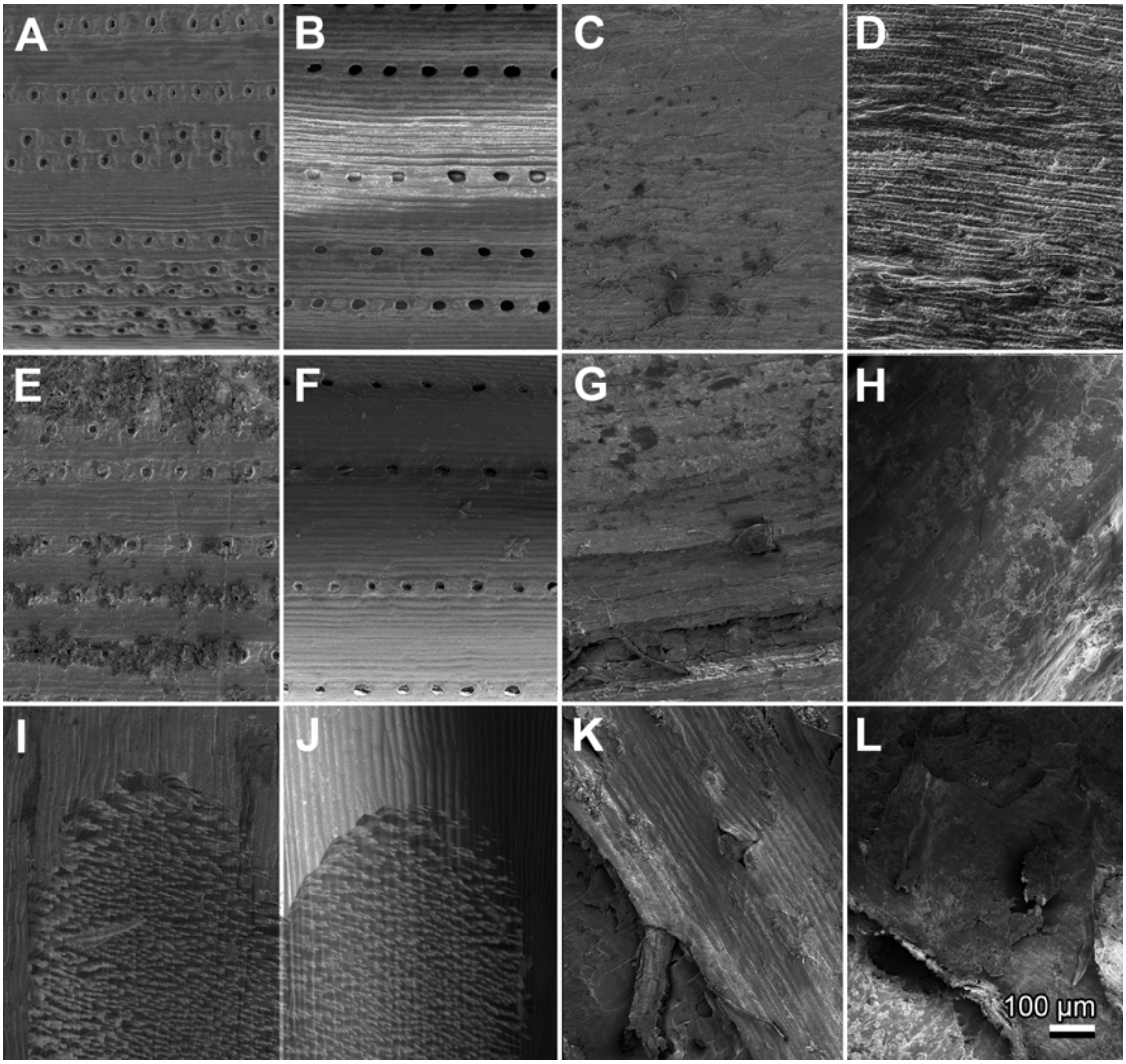

Fig. S2 The surface host plants of *Monochamus alternatus*, 4-5-years-old seedlings of *Pinus densiflora* (A, C, E, G, I, K.) and *Pinus thunbergii* (B, D, F, H, J, K). **A, B.** Needles of current-year shoot. **C, D.** Bark of a branch. **E, F.** Needles of one-year-old shoot. **G, H.** Bark of the upper trunk. **I, J.** Shafts of needles of current-year shoot. Note the parallel surface folds. The insets indicate the bilobed 3rd tarsomere of a female foreleg, for comparison of dimensions. **K, L.** Bark of the lower trunk.

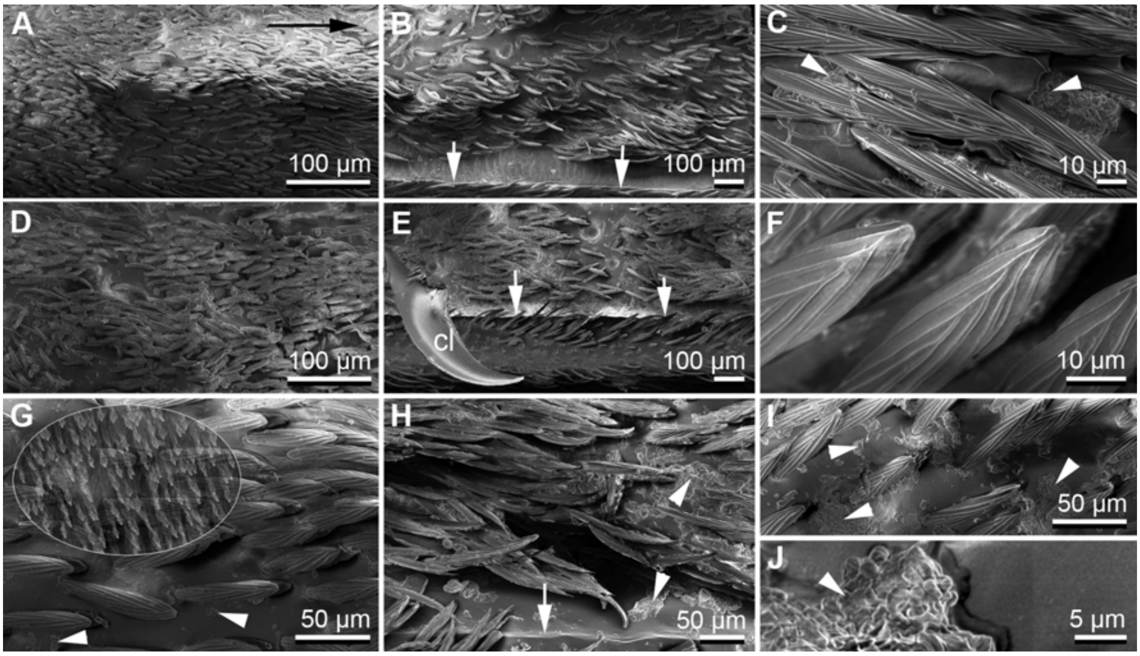

Fig. S3 The elytra surface of a female *Monochamus alternatus* is covered with microtrichia; between them hair-free patches are found. The black arrow in A points distally. **A.** Dorsal, thoraco-abdominal view. **D.** Lateral, thoraco-abdominal view. **G.** Detail of short microtrichia in comparison with the dimensions of spatula-shaped adhesive setae (inset). **B, E, F.** Arrows point to the sharp edge at the lateral margin of elytra, in comparison with a part of the claw (cl, inset in E), which is interlocking at that site during copulation. Along the sharp edge, a hair-free line is running along the elytra. **C, F, I, J.** Details of microtrichia, longer ones (C), shorter ones (F); and the grease (arrow heads), which partly covers the microtrichia and hair-free patches.

Contact angle measurements on host substrates

Wettability and free surface energy (FSE) can significantly influence insect attachment, mostly by promoting or preventing the wetting with tarsal adhesion-mediating secretion (Gorb and Gorb, 2009; Dirks and Federle, 2011; Grohmann et al., 2014). That is why static contact angles of well-characterized liquids Aqua Millipore water, diiodomethane (DCG6885, Wako Pure Chemical Industries, Ltd., Japan), ethylene glycol (TLG0583, Wako Pure Chemical Industries, Ltd., Japan) on the pine surfaces, female elytra, and control glass were measured (free sessile drop method with ellipse-fitting). Therefore, cut pieces (5 x 10 mm) per each, seedling from (a) bark of lower trunk, (b) bark of middle trunk, (c) bark of upper trunk, (d) 10-mm long needles of one-year old shoot, (e) 10-mm long needles of current year shoot, (f) female elytra, (g) pre-cleaned micro glass slides (thickness 0.9~1.2 mm, 76 x 26 mm, S-7213, Matsunami glass Ind., Ltd., Japan), (h) a pre-cleaned, custom-made glass plate, and (i) pre-cleaned, custom-made, 10-cm long, 2-cm wide glass tubes. Prior the tests, glasses were successively rinsed with aceton, ethanol and Aqua Millipore water, and immediately dried with compressed air. Samples were attached evenly with the adaxial side up on a clean glass slide by double-sided adhesive tape. Using a pipette Eppendorf Reference 0.1-2.5 µl and pipette tips (ep T.I.P.S. 0.1-10 µl A145595R) (Eppendorf AG, Hamburg, Germany), drops (1 µl) of three liquids were applied on each test surface and simultaneously visualized with a horizontally aligned motion analysis microscope VW-6000, combined with high-performance low-range zoom lens VH-Z00R, at a magnification of 50x and 80 fps (Keyence Corp., Tokyo, Japan) at 22.3 ± 2.52 °C temperature and 29.1 ± 6.15% relative humidty.

From obtained images, ten contact angle measurements with each fluid on each surface, in total, 270 single contact angle measurements were performed using the SCA 20 Demo software (DataPhysics Instruments GmbH, Filderstadt, Germany). The FSE of test surfaces was calculated according to the OWRK method (Owens and Wendt 1969).

References

Gorb E., Gorb S. 2009. Effects of surface topography and chemistry of *Rumex obtusifolius* leaves on the attachment of the beetle *Gastrophysa viridula*. Entomologia Experimentalis et Applicata 130: 222-2228.

Dirks J.-H., Federle W. 2011. Mechanisms of fluid production in smooth adhesive pads of insects. Journal of the Royal Society Interface 8: 952–960.
Grohmann C., Blankenstein A., Koops S., Gorb S. N. 2014. Attachment of *Galerucella nymphaeae* (Coleoptera, Chrysomelidae) to surfaces with different surface energy. Journal of Experimental Biology 217: 4213-4220.
Owens D. K., Wendt R. C. 1969. Estimation of the surface free energy of polymers. Journal of Applied Polymer Science 13: 1741-1747.

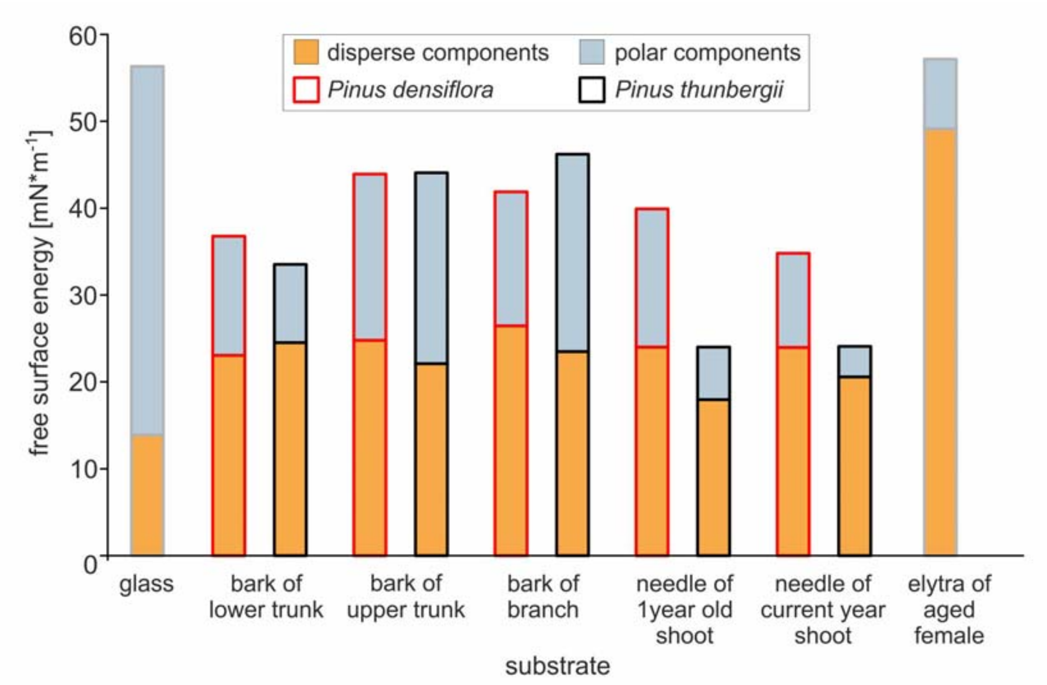

Fig. S4 Total free surface energy (FSE) of various surfaces and its partition into disperse and polar components. Examples of natural substrates of *Monochamus alternatus* (bark and needles of two pine species, elytra of female *M. alternatus*) are compared with cleaned, normal glass.

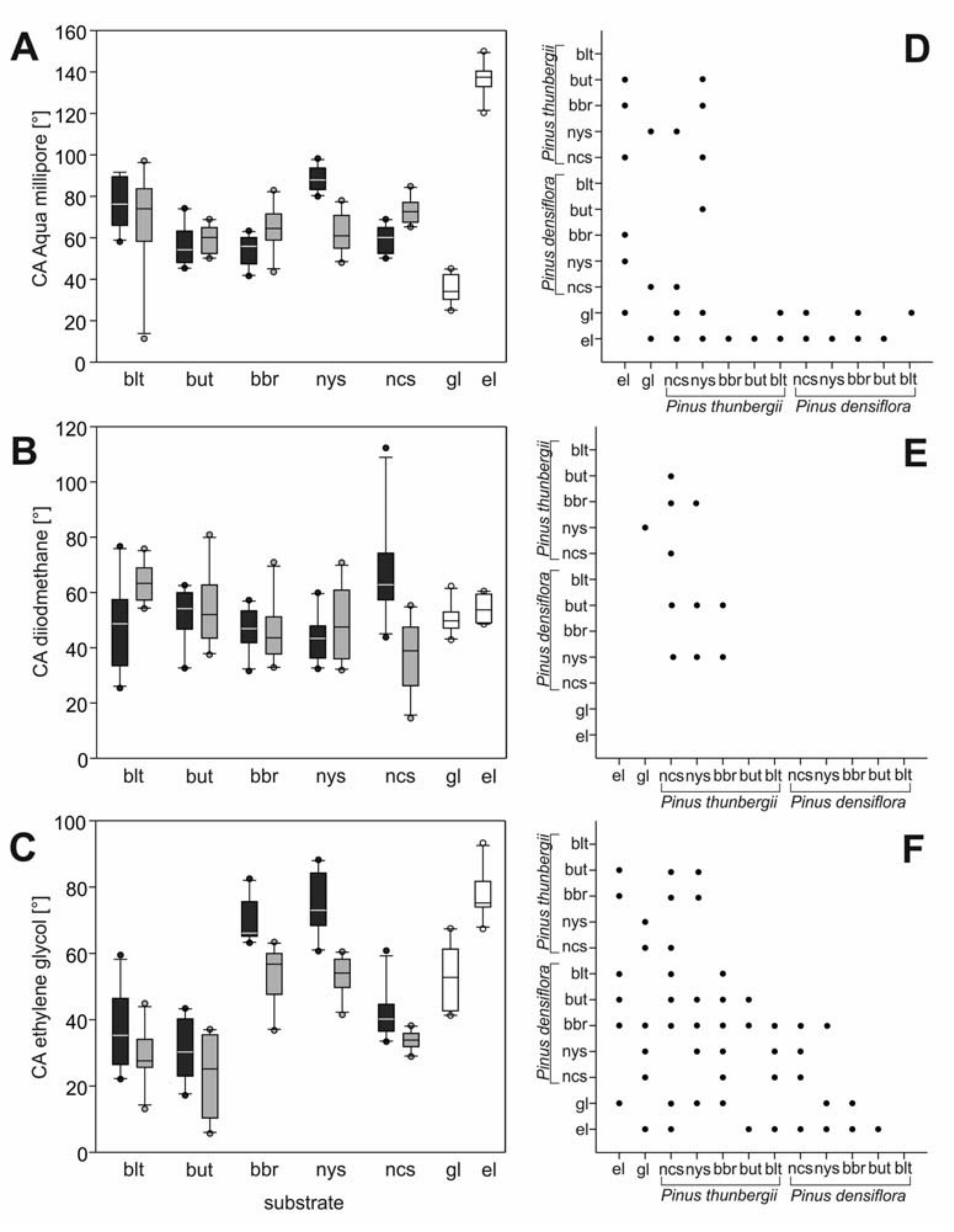

Fig. S5 Contact angles [°] (**A-C**) of water (A, D), diiodomethane (B, E), and ethylene glycol (C, F) to glass, pine and elytron surfaces. **D**–**F**. Plots showing the results of pairwise multiple comparisons between surfaces (Kruskal-Wallis one-way ANOVA on ranks followed by Tukey test at $p < 0.05$; $H_{11,118} = 83.9$, $p \leq 0.001$ for water (A); $H_{11,118} = 42.3$, $p \leq 0.001$ for diiodomethane (B), and $H_{11,118} = 98.4$, $p \leq 0.001$ for ethylene glycol (C); black dots indicate significant differences in contact angles between tested surfaces (B, D, F). blt, bark of lower trunk; but, bark of upper trunk; bbr, bark of an 1-cm thick branch; CA, contact angle; el, elytra; gl, glass; ncs, needle of current shoot; nys, needle of 1year-old shoot.
